# A novel mutation in the *ADA* gene causing severe combined immunodeficiency in an Arab patient: a case report

**DOI:** 10.1186/1752-1947-3-6799

**Published:** 2009-04-01

**Authors:** Ali Hellani, Nidal Almassri, Khaled K Abu-Amero

**Affiliations:** 1 PGD Laboratory, Saad Specialist Hospital, Al-khobar, 31952, Saudi Arabia; 2 Pathology and Laboratory Medicine Department, Saad Specialist Hospital, Al-khobar, 31952, Saudi Arabia; 3 Molecular Genetics Laboratory, College of Medicine, King Saud University, PO Box 245, Riyadh 11411, Saudi Arabia

## Abstract

**Introduction:**

About 20% of the cases of human severe combined immunodeficiency are the result of the child being homozygous for defective genes encoding the enzyme adenosine deaminase. To our knowledge, the mutation pattern in Arab patients with severe combined immunodeficiency has never been reported previously.

**Case presentation:**

A 14-month-old Arab boy had clinical features typical of severe combined immunodeficiency. His clinical picture and flow cytometric analysis raised the diagnosis of adenosine deaminase deficiency and prompted us to screen the adenosine deaminase gene for mutation(s). We detected a novel mutation in exon 9 of the adenosine deaminase gene (p.Arg282>Gln), which we believe is the cause of the severe combined immunodeficiency phenotype observed in our patient.

**Conclusion:**

This is the first report of adenosine deaminase mutation in an Arab patient with severe combined immunodeficiency due to a novel pathogenic mutation in the adenosine deaminase gene.

## Introduction

Human severe combined immunodeficiency (SCID) was first reported by Swiss workers more than 50 years ago [1]. Infants with the condition were profoundly lymphopenic and died of infection before their second birthday. In the ensuing years, differences in inheritance patterns were noted, indicating causal heterogeneity of this condition. In many families, there is a clear X-linked pattern of inheritance, whereas in others there is an autosomal recessive inheritance pattern. The first discovered molecular cause of human SCID, adenosine deaminase (ADA) deficiency, was reported in 1972 [2]. Infants with this syndrome are lymphopenic and have profound deficiencies of T and B cell numbers and function. Adenosine deaminase is an important deaminating enzyme, which converts adenosine and 20-deoxyadenosine to inosine and 20-deoxyinosine, respectively. Those components are generated in large amounts from apoptotic cells in the thymus, marrow, and lymph nodes [3]. When ADA is absent, these nucleosides are metabolized differently and have distinct biochemical actions. The incidence of ADA deficiency is about 1 in 1,000,000 births, but it accounts for 10% to 20% of all cases of SCID. In about a fifth of ADA-deficient patients, the immune deficiency is initially less severe, presenting later in childhood. A few older patients have recently been diagnosed at 15-40 years of age with chronic pulmonary insufficiency due to recurrent respiratory infections, and other manifestations of immune deficiency and dysregulation [4].

The genomic sequence of ADA gene spans 32kb on the long arm of chromosome 20 and contains 12 exons [5]. More than 70 ADA mutations have been identified thus far. The majority were missense mutations (63%), 18% were splicing mutations, 13% were deletions and 6% were nonsense mutations [6]. The amino acid substitutions are distributed throughout the protein sequence. A few large deletions have arisen from recombination between Alu repeats, but most deletions are small and probably due to replication errors. Most recurrent missense mutations arise from codons that contain the CpG dinucleotide. This report describes the identification of a novel base-substitution mutation in the ADA gene along with the associated phenotype, and the modalities of treatment related to gene findings.

## Case presentation

A 14-month-old Arab boy whose parents were first-degree cousins was referred to our pediatric ward with a history of frequent severe chest infections, chronic diarrhea and failure to thrive. This family lost two children at the age of 6 and 8 months due to SCID. The patient was found to be lymphopenic and further investigations confirmed severe combined immunodeficiency with the first sign being fever at 1 month of age. The cause of his immunodeficiency was suspected to be ADA deficiency. He also had a Mycobacterium intracellulare infection and was suspected of having cytomegalovirus (CMV) gastroenteritis. He was irritable, in compensated sepsis with respiratory distress and his abdomen was soft with no palpable hepatosplenomegaly. He had a fine skin rash with no lymphadenopathy and his BCG vaccination site showed a non-infected old scar. The rest of his physical exam was normal. His past medical history revealed recurrent severe infections, especially chest infections. He had had at least four severe episodes of lower respiratory tract infection with bronchospasm. Almost all of these episodes needed admission to hospital and treatment with intravenous antibiotics. History of the index case also included diarrhea with loose stools 4 to 5 times daily. At 1 month of age, he developed a bacterial infection easily cured with a course of antibiotics. Investigations revealed mild hydro-uretero-nephrosis and no evidence of vesico-ureteric reflux. Past surgical history included a left inguinal hernia repair. Flow cytometry (FCM) analysis was performed in order to quantify the number of B- T-, and NK-cells. FCM results showed near absences of lymphocytes with only 101 cells per μl (2% of white blood cells). The lymphocyte subsets were 88% T cells with a CD4 to CD8 ratio of 3; 5% B-cells and 7% NK-cells. Absence of the common γ chain (CD132) which is associated with X-linked SCID was not evaluated. However, unlike our patient, X-linked SCID patients characteristically have a T-B+NK-phenotype which is different from our patient who has a T-B-NK-phenotype. The latter phenotype is seen in ADA deficiency [7] and hence prompted our evaluation and study of the ADA gene mutation.

In order to assess the mutation causing SCID in this family, genomic DNA was amplified and the entire coding region and the exon-intron boundaries of the ADA gene were sequenced. The primers used to amplify all 12 exons of the ADA gene are summarized in Table 1. Briefly, ADA sequences were amplified from 100 to 200ng of DNA using specific primers (5μMdNTP (5mM), PCR buffer 10×, and one unit of expanded long Taq polymerase (Roche). Polymerase chain reaction (PCR) products were purified using a Qiagen purification kit and then assessed with a capillary electrophoresis bio-analyzer using the DNA 7500 chip. The purified PCR products were sequenced on an ABI 3130xI Genetic Analyzer using forward and reverse primers listed in Table 1.

**Table 1:** List of primers covering the entire exons and intron-exon boundaries for the *ADA* gene

Exon	Primer sequence	Tm (°C)	Size (bp)
1	F-5′-TGTGTGTTTCTGCGACGAGC-3′	55	692
	R-5′-TGTCCCTGATTAGCCCGCAA-3′		
2	F-5′-GCAGCCAGCCAGTAAAATG-3′	55	366
	R-5′-TGTCCTCACAGTCCCACTTC-3′		
3	F-5′-GTCCACCACTCACTGTTTTG-3′	55	384
	R-5′-AGTCCATCACACCCACATC-3		
4	F-5′-TGTTCCCAACCCCTTTCTTCC-3′	55	556
	R-5′-AAATGGGCCAGACTCACTTCAG-3′		
5	F-5′-CCCAAAGCCTCCTCTTCCTCCT-3'	55	377
	F-5′-AGGTCTCCAGTTGTTTCATG-3′		
6	F-5′-TAGGCTGGGAGGTCTCTC-3′	55	315
	R-5′-ACCCAACAAAGACACACTC-3′		
7-9	F-5′-ATGCTGTTGAAGCAGGCAGCATGACTAGGA-3'	60	739
	F-5′-TGCCTGCTTCCCAGGGTGTCGAAGAGATTT-3′		
10	F-5′-AGGATCAAAGGCGGGTGAAC-3′	55	312
	R-5′-TCCCTCTCTCCAAAGATTCCAG-3′		
11	F-5′-AGGATCAAAGGCGGGTGAAC-3′	55	238
	R-5′-TCCCTCTCTCCAAAGATTCCAG-3′		
12	F-5′-TCTGAAGCCCAGTCCCAAAG-3′	55	363
	R-5′-AAATGTTGCTCAGCCCCAC-3′		

Corresponding Tm and size in bp are indicated. PCR conditions as described in the case presentation.

Predictions of the tolerance of the protein to the mutation were assessed using SIFT (Sorting Intolerant From Tolerant) programs (http://blocks.fhcrc.org/sift/SIFT.html) and Protean (Protein Structure Prediction and Annotation; LASERGENE V.6 software DNASTAR, Inc., Madison, WI, USA). Protean helps to predict and display patterns, secondary structural characteristics and physiochemical properties (hydropathy index and flexibility prediction). The study has been reviewed by the Evidence Based Medicine and Research center of this institution.

**Figure 1:**
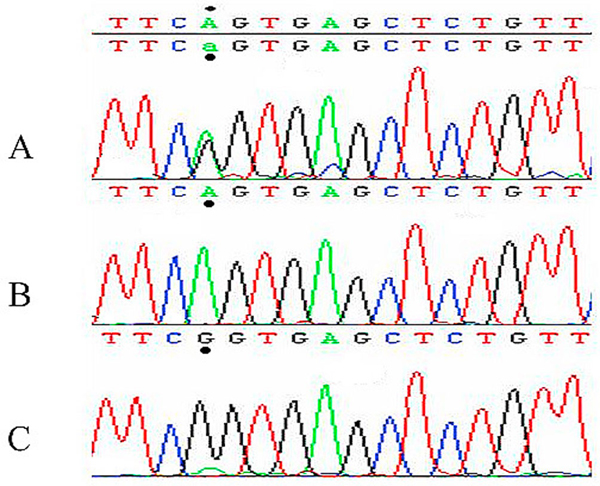
**Chromatogram presenting the sequence of exon 9 of the *ADA* gene, where the mutation was detected**. The mutation involves the substitution of nucleotide G by A. The parents and sister **(A)** are heterozygous for this mutation; the affected child **(B)** is only showing nucleotide A while in normal DNA **(C)** only nucleotide G is present. The nucleotide changes are marked.

After sequencing the entire ADA gene, we detected a single nucleotide substitution (c.847G>A) in exon 9 of this gene (Figure 1). This nucleotide substitution resulted in the replacement of Arginine (Basic hydrophilic AA) with Glutamine (Neutral AA) at codon 282 (p.Arg282>Gln) thus altering the hydropathy index from 0.48 to 0.37. Protean predicted a change in the amino acid structure and this may affect the function. The mutation was located in the active domain of the protein, which extends from codon 8 to 346, thus it was expected to affect the deaminase activity of the enzyme. SIFT predicted that the AA change at this location could not be tolerated, and thus it is highly likely that it will have an effect on the protein structure and/or function. This sequence change was not detected in 50 unrelated controls of similar ethnicity. Based on the above findings, we strongly believe that this sequence change is probably pathogenic.

## Discussion

The mutation is a G to A transition at a CpG dinucleotide, CGG to CAG, and is therefore a "hotspot" mutation, as over half the missense mutations are at the ADA locus [8]. Methylated cytosine can spontaneously deaminate to thymine, resulting in a C to T transition if the deamination initially occurs on the coding strand and a G to A transition if the alteration initially occurs on the non-coding strand. Since methylation of cytosine in man occurs virtually exclusively at CpG dinucleotides, CpG dinucleotides are hotspots for mutations. Despite being a hotspot mutation, the mutation identified in this report is novel. Moreover, this is the first report of a mutation in exon 9 of the ADA gene where mutation(s) are rarely present [9,10]. We believe that this mutation is the cause of the SCID phenotype observed in our patient for the following reasons: i) it is the only mutation found after sequencing the entire ADA gene; ii) both parents were heterozygous for this mutation; iii) the affected individual was homozygous for this mutation; iv) the location of the mutation and the SIFT prediction; v) alterations in the hydropathy index and vi) the fact that this mutation was not found in 50 individuals (100 alleles) from matching ethnicity.

The effects of mutation on the ADA would be more precisely assessed by measuring, i) the level of soluble ADA activity and ii) the immunoreactive ADA protein expressed by ADA cDNAs in *E. coli*, which has a deletion of the bacterial ADA gene [11]. The concentration of wild-type ADA and the activity of the mutated enzyme are expected to be extremely low since SCID is present in all of the affected members of the family. Indeed, Hershfield suggested that patients whose two alleles together provide ≤0.01% of wild-type ADA activity have SCID; those with 0.1% to 0.3% have delayed or late onset SCID; Furthermore, those inheriting alleles that provide ≥5% of wild-type activity remain healthy [3]. Three types of therapy are available for patients suffering from ADA deficiency: bone marrow transplant, enzymic treatment and gene therapy. Hematopoietic stem cell transplantation (HSCT) from a human leucocyte antigen (HLA) -matched donor offers good immunological and biochemical correction with survival ca.85%, but in the mismatched donor setting, the outcome is significantly worse. Pegademase (PEG)-ADA enzyme replacement therapy is used when a matched donor is unavailable. Although overall survival is good [12], immune reconstitution is variable and prolonged treatment results in significant lymphopenia, variable defects in T cell function including decreased thymic output, and susceptibility to viral infection. Initial retroviral vector-mediated gene therapy trials for ADA-SCID demonstrated efficient transduction of hematopoietic progenitors and long-lived expression of the ADA gene in transduced T-lymphocytes, but immune reconstitution was poor and most probably compromised by the concomitant use of PEG-ADA, which abrogated the survival and growth advantage of gene-modified cells [13]. The success of gene therapy for ADA-SCID has recently been shown in patients who did not commence PEG-ADA and who received a non-myeloablative chemotherapy regimen [14]. It is likely that the lack of PEG-ADA restores a survival advantage to transduced lymphocytes and that chemotherapy allows increased engraftment of gene-transduced progenitors. The patient described in this report is benefiting from the HSCT program where his sister, a carrier for the currently reported mutation, is a matching donor. The family is benefiting as well from our preimplantation genetic diagnosis [15,16] facility where a future pregnancy would be free of ADA deficiency.

## Conclusions

The promising success of hematopoietic stem cell transfer and gene therapy in patients with adenosine deaminase deficiency provides tremendous hope for the patients and their families. Identifying adenosine deaminase gene mutation(s) involved in the severe combined immunodeficiency is a key element for any subsequent treatment. This is the first report of adenosine deaminase mutation in an Arab patient with severe combined immunodeficiency.

## Abbreviations

ADA: adenosine deaminase; CMV: cytomegalovirus; FCM: flow cytometry; HLA: human leucocyte antigen; HSCT: hematopoietic stem cell transfer; PCR: polymerase chain reaction; PEG: pegademase; SCID: severe combined immunodeficiency; SIFT: sorting intolerant from tolerant.

## Consent

Written informed consent was obtained from the parents of the patient for publication of this case report and any accompanying images. A copy of the written consent is available for review by the Editor-in-Chief of this journal.

## Competing interests

The authors declare that they have no competing interests.

## Authors' contributions

AH AND KKA drafted and revised the manuscript. All authors have read and approved the final manuscript. AH was in charge of design, analysis of data and overall supervision of the study. KKA performed the technical aspects of the study, PCR and sequencing. NA was in charge of the flow cytometry analysis and interpretation of the routine laboratory results.
